# Dual-Acting Compounds Targeting Endocannabinoid and Endovanilloid Systems—A Novel Treatment Option for Chronic Pain Management

**DOI:** 10.3389/fphar.2016.00257

**Published:** 2016-08-17

**Authors:** Natalia Malek, Katarzyna Starowicz

**Affiliations:** Laboratory of Pain Pathophysiology, Department of Pain Pharmacology, Institute of Pharmacology, Polish Academy of SciencesKrakow, Poland

**Keywords:** FAAH, TRPV1, COXs, endocannabinoid system, dual acting compounds, pain

## Abstract

Compared with acute pain that arises suddenly in response to a specific injury and is usually treatable, chronic pain persists over time, and is often resistant to medical treatment. Because of the heterogeneity of chronic pain origins, satisfactory therapies for its treatment are lacking, leading to an urgent need for the development of new treatments. The leading approach in drug design is selective compounds, though they are often less effective and require chronic dosing with many side effects. Herein, we review novel approaches to drug design for the treatment of chronic pain represented by dual-acting compounds, which operate at more than one biological target. A number of studies suggest the involvement of the cannabinoid and vanilloid receptors in pain. Interestingly cannabinoid system is in interrelation with other systems that comprise lipid mediators: prostaglandins, produced by COX enzyme. Therefore, in the present review, we summarize the role of dual-acting molecules (FAAH/TRPV1 and FAAH/COX-2 inhibitors) that interact with endocannabinoid and endovanillinoid systems and act as analgesics by elevating the endogenously produced endocannabinoids and dampening the production of pro-inflammatory prostaglandins. The plasticity of the endocannabinoid system (ECS) and the ability of a single chemical entity to exert an activity on two receptor systems has been developed and extensively investigated. Here, we review up-to-date pharmacological studies on compounds interacting with FAAH enzyme together with TRPV1 receptor or COX-2 enzyme respectively. Multi-target pharmacological intervention for treating pain may lead to the development of original and efficient treatments.

## Introduction

Pain is defined by the International Association for the Study of Pain as an unpleasant sensory and/or emotional experience associated with actual or potential tissue damage (Bonica, 1979). Acute pain is important for the diagnosis and localization of the disease process and to avoid or minimize tissue damage. In a situation where the pain sensation remains despite mitigating the immediate cause, it loses its warning and defensive characteristics and becomes a disease itself, which is challenging to treat because of the heterogeneity of its origin. None of the currently available therapies provide sufficient relief for patients; at the same time, they all have risks of complications and a number of adverse effects as incidence increases with age (Ray et al., 2007; Gallagher and Rosenthal, 2008; Williams et al., 2013). Due to the aging population in developed countries and the lack of satisfactory therapies, chronic pain is a worldwide disease that requires the development of new treatments. One possible approach focuses on the modulation of endogenously produced compounds. The lipophilic molecule anandamide (AEA), that is an integral part of the endocannabinoid system (ECS), also binds to the transient receptor potential cation channel subfamily V member 1 (TRPV1); consequently, AEA is now frequently referred to as an “endovanilloid.” A promising approach to retain the analgesic effects of cannabinoid activation, while avoiding the undesirable results of its global action is to elevate endogenously produced endocannabinoids by inhibiting their hydrolytic degradation. Fatty acid amide hydrolase (FAAH) hydrolyzes AEA and other lipid signaling molecules, which makes it a good target for this new therapeutic method. Moreover, ECS is interrelated with other systems that comprise lipid mediators such as prostaglandins/leukotrienes, contributing to the chronic pain development (Huwiler and Pfeilschifter, 2009). Additionally, compounds with primary actions upon cyclooxygenase (COX), involved in pain processing, also interact with ECS, including having the ability to directly inhibit FAAH (Fowler, 2007; Guindon and Hohmann, 2008).

**Figure 1:**
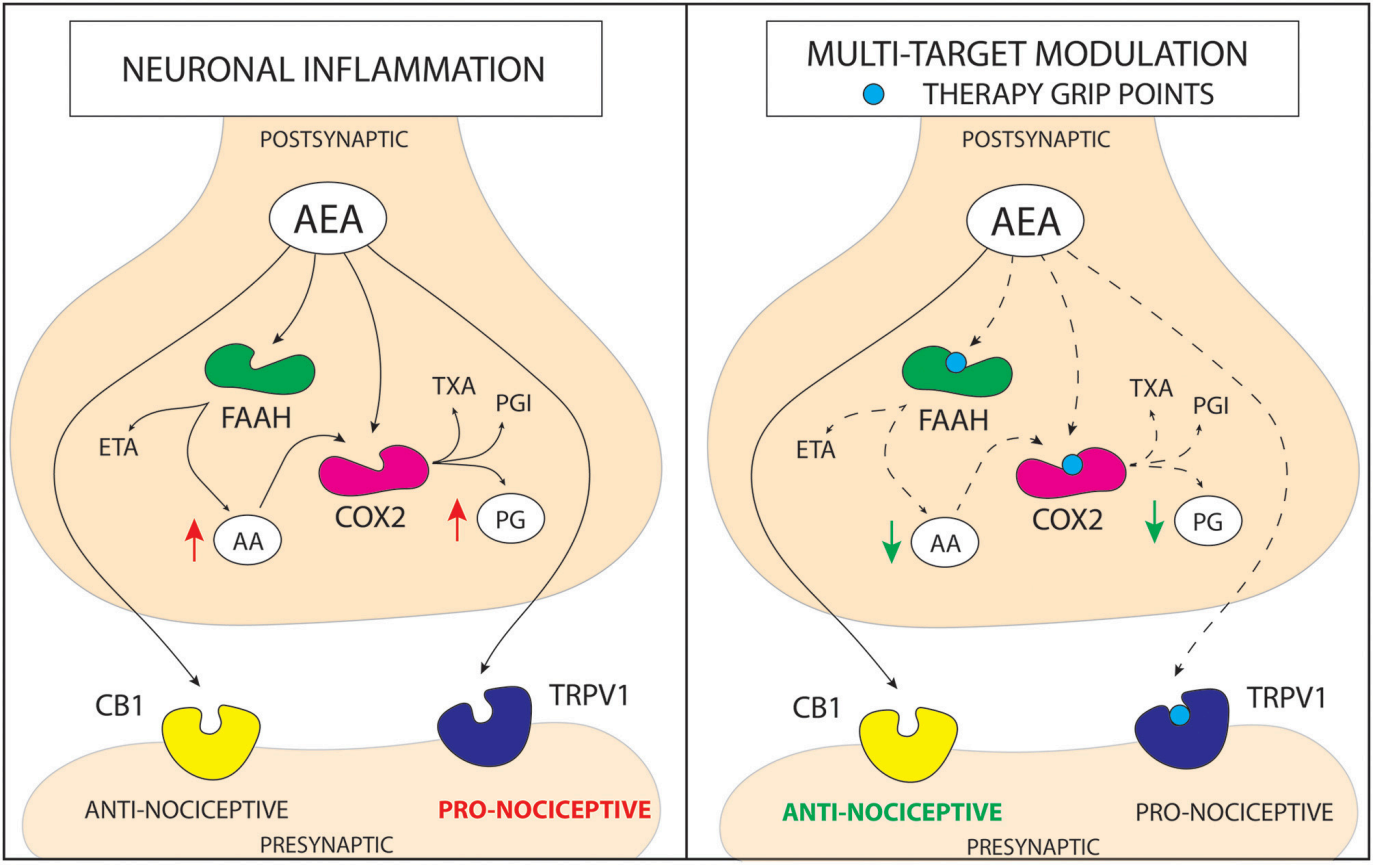
**Schematic representation of a mechanism of action for cannabinoid based multi-target drugs**. The novel strategy for the pain treatment is based on altering more than one enzymatic reaction and/or nociceptive pathway. Anandamide (AEA) is hydrolyzed into arachidonic acid (AA) and ethanolamine (ETA) principally by fatty acid amide hydrolase (FAAH). In addition to its hydrolysis by FAAH, AEA is metabolized by COX-2. On the other hand, COX metabolizes AA, leading to increase in pro-inflammatory prostaglandins (PG). AEA can act on transient receptor potential cation channel subfamily V member 1 (TRPV1) activating pro-nociceptive pathways or through cannabinoid receptor 1/2 (CB1/2) pathway leading to alleviation of pain. Although, in the molecular stress conditions elevation of AEA occurs, FAAH and COX quickly metabolize it. Therefore, there are three pathways that may increase anti-nociceptive properties of AEA; (1) inhibition of FAAH enzyme, that leads to an increase in AEA level and a decrease in AA level; (2) inhibition of COX enzyme, that leads to an increase in AEA level and a decrease in PG level; (3) antagonism of TRPV1 receptor, which prevents activation of pro-nociceptive pathway by AEA. Action on more than one molecular target lowers the redundancy of the system and may lead to obtaining more stable and robust response in pain alleviation. (PGI—prostacyclins; TXA—thromboxanes).

Recent studies suggest that inhibiting FAAH will not have as significant efficacy as expected in chronic pain patient groups (Huggins et al., 2012). Therefore, it could be more relevant for future research to take into account a new paradigm—a multi-therapeutic strategy combining the action on FAAH, TRPV1, and COX-2. Here, we review up-to-date studies on dual-acting compounds that interact with the endocannabionid/endovanilloid and COX systems, which may be beneficial for the treatment of chronic pain (the basis of molecular interactions between the systems is shown in Figure 1).

## Single target: success or defeat?

### CB_1_ and CB_2_ agonism

In the recent years, considerable progress has been made in understanding the role of the ECS in the modulation of pain processing. The system includes cannabinoid receptors 1 (CB_1_) and 2 (CB_2_), endogenous agonists called endocannabinoids: AEA and 2-arachidonyl glycerol (2-AG) and enzymes involved in their biosynthesis and degradation. Endocannabinoids are produced in injured tissues to suppress sensitization and inflammation by activation of CB_1_ and CB_2_ (Piomelli and Sasso, 2014). The key enzyme responsible for the catabolism of AEA is FAAH. However, AEA can also be metabolized by COX-2 and lipoxygenase 12/15 (LOX12/15). COX-2 metabolizes AEA to prostaglandins (PG), which are involved in inflammatory response (Kozak et al., 2002; Yang et al., 2005; Fowler, 2007). CB_1_ receptors expressed in the peripheral and central nervous system (CNS) are responsible for the conducting of pain signaling (Richardson et al., 1998). CB_2_ receptors expressed in immune cells (also microglia), regulate the neuro-immune interactions and interfere with the inflammatory hyperalgesia, thus also playing an important role in nociception (Malek et al., 2015b; Mecha et al., 2015). Endocannabinoids have been shown to behave as analgesics in models of both acute nociception and chronic pain such as inflammation (Pernía-Andrade et al., 2009) and painful neuropathy (Toth et al., 2010; La Porta et al., 2013). The main limitation of exogenous cannabinoid receptor agonists is the number of CNS side effects, including dysphoria, movement disorders, dizziness, memory loss, drug abuse potential, and dependence (Thaler et al., 2011). An alternative approach that may prevent the occurrence of these side effects is the stimulation of endogenous mechanisms that regulate ECS activity (Lichtman and Chapman, 2011; Lomazzo et al., 2015).

### FAAH inhibition

Endogenous cannabinoids are synthesized “on demand” in areas of cellular stress and therefore do not produce CB_1_ agonist side effects (Di Marzo and Petrosino, 2007). Unfortunately, locally released endocannabinoids have a very short half-life due to efficient enzymatic degradation (Deutsch and Chin, 1993; Ahn et al., 2008). Therefore, a subsequent grip point for pain therapies is FAAH, which is responsible for the degradation of AEA and other related fatty acid amides including N-palmitoylethanolamine (PEA), oleoylethanolamine (OEA), N-arachidonoyl glycine (NAGly; Bisogno et al., 2002; Khanna and Alexander, 2011). A series of *in vivo* experiments proved that genetic or pharmacological inactivation of FAAH results in elevation of endocannabinoids in the spinal cord and brain stem (Lichtman et al., 2004; De Lago et al., 2005). FAAH inhibition, rather than its deletion, may offer a distinctive strategy for the treatment of chronic pain, because no genotypic differences in pain behavior were evident in chronic pain models (Lichtman et al., 2004; Kinsey et al., 2009). FAAH inhibition was shown to cause anti-nociceptive, anti-inflammatory or anti-edemic effects in numerous acute (Holt et al., 2005), chronic (Jayamanne et al., 2006), and neuropathic pain animal models (Jhaveri et al., 2006; Kinsey et al., 2009; Guindon et al., 2013). Nevertheless, FAAH inhibitors (like URB957) are not optimal due to some limitations. Tissue-specific changes in the sensitivity to URB597 in neuropathic pain in rats, which may arise as a result of changes in FAAH activity, metabolic pathways, and tissue pH were reported (Chang et al., 2006; Paylor et al., 2006). Due to the differential effects of URB597 in carrageenan-induced inflammation and spinal nerve ligation models, efforts aimed at optimizing the clinical efficacy of FAAH inhibitors should be revised and redesigned (Di Marzo, 2012; Okine et al., 2012). Similarly, despite many promising preclinical results in various chronic pain models (Ahn et al., 2011), one of the most potent FAAH inhibitors, PF-04457845, has failed to show efficacy in humans in a randomized, placebo-controlled phase II clinical trial (Di Marzo, 2012; Huggins et al., 2012). Moreover, safety of FAAH inhibitors became questioned after “first-in-human” trial to test safety of BIA 10-2474 in healthy volunteers, which concluded with one person dead and five more hospitalized. The probable cause of the failure is unjustified dose, 80 times higher than that presumed to induce total FAAH inhibition, used in the study^1^.

### TRPV1 antagonism

TRPV1 has emerged as a promising target for the development of new analgesic and anti-inflammatory drugs. TRPV1 is a non-selective ion channel that is highly associated with pain nociception and linked to ECS through the common agonist AEA (Zygmunt et al., 1999; Van der Stelt et al., 2005; Lizanecz et al., 2006). This polymodal pain transducer is known to be expressed in peripheral sensory afferents (Singh Tahim et al., 2005; Ikeda-Miyagawa et al., 2015), spinal cord (Kanai et al., 2006; Horvath et al., 2008), and some brain stem nuclei involved in nociception, including periaqueductal gray matter (PAG) and cingulate cortex (Roberts et al., 2004; Cristino et al., 2006; Starowicz et al., 2007).

A growing body of evidence suggests that TRPV1 is essential in driving nociceptive response (Davis et al., 2000; Immke and Gavva, 2006; Horvath et al., 2008). TRPV1-knockout mice exhibited attenuated inflammation-induced hyperalgesia, while receptor blockade and desensitization caused analgesia (Karai et al., 2004; Bölcskei et al., 2005). Furthermore, enhanced TRPV1 expression and increased TRPV1 sensitivity to AEA during inflammation and neuropathic conditions has been described previously (De Petrocellis et al., 2001; Baamonde et al., 2005; Singh Tahim et al., 2005). Unfortunately, off-target effects of TRPV1 modulation on thermoregulation are widely described. TRPV1 activation results in hypothermia, whereas antagonizing TRPV1 causes hyperthermia, which accounted for the failure of AMG517 during a phase I clinical trial (Di Marzo et al., 2000; Swanson et al., 2005; Gavva et al., 2008). Nevertheless, TRPV1 antagonism has still emerged as an interesting strategy to alleviate pain, especially with simultaneous action on either FAAH and/or COX-2, which could allow lower doses to be used (Lee et al., 2015; Malek et al., 2015a, 2016).

### COX-2 antagonism

The basis for the use of non-steroidal anti-inflammatory drugs (NSAIDs) for the treatment of pain and inflammation is COX inhibition. Cyclooxygenases are enzymes that catalyze the conversion of membrane phospholipids to prostanoids, which include PG, prostacyclins (PGI) essential for intestine and kidney functioning, and thromboxanes (TXA), responsible for platelet aggregation. The two COX isozymes are COX-1 and COX-2. COX-1 is characterized by constitutive expression in tissues and COX-2 is an induced isoform with low baseline expression in human tissues that can be induced during a response to extracellular and intracellular stimuli. The increase in COX-2 expression elevates proinflammatory cytokine, mitogen and growth factor levels (Smith et al., 1996), as well it plays a key role in the transmission of pain to the brain and spinal cord (Ito et al., 2001). Moreover, blocking COXs can influence the ECS by stopping the conversion of endocannabinoids into prostanoid-like derivatives, which may be of benefit for the treatment of chronic pain (Jhaveri et al., 2007; Hu et al., 2008). The inflammatory process can dramatically reduce the level of endocannabinoids, while its inhibition may contribute to increased analgesic action. Although widely used in the treatment of pain, NSAIDs do not always provide adequate relief and have the risk for serious complications, including death by internal hemorrhaging (5–10% of chronic NSAIDs therapy) and severe kidney damage (Rahme and Bernatsky, 2010; Altman, 2011). However, it should be noted that some of the NSAIDs have the ability to inhibit FAAH (Fowler et al., 1999; Seidel et al., 2003; Guindon et al., 2006). Therefore, interaction of NSAIDs with ECS may be a novel approach for the treatment of chronic pain.

## Two targets: improvement or deterioration?

The aging population in developing countries, the lack of satisfactory therapies, and worldwide increase of chronic pain are burdens that require the development of new treatments for pain. A leading approach in the treatment of chronic pain is using single-target drugs, which are not always effective and can be associated with many side effects from chronic dosing. Multi-functional drugs may be advantageous for poorly controlled chronic pain and may improve safety and efficacy properties with fewer side effects compared to single-target molecules (Csermely et al., 2005; Stahl, 2009). Dual-acting compounds also have much more potential than single-target and highly specific agents due to better safety profiles and are less likely to exhibit pharmacokinetic and pharmacological interactions (compared to combined therapy with two single-target drugs). With respect to pain relief, there are some reports about multi-target therapeutics that will be discussed here, including piperazinyl carbamates that simultaneously interact with FAAH and TRPV1 or FAAH and COX inhibitors. We will determine the status of *in vivo* preclinical drug research and consider whether multi-target drugs are the current trend in search for better pain therapy (summarized information on the dual-acting compounds can be found in the Table 1).

**Table 1:** **Dual-acting compounds: summary of ***in vivo*** studies**.

**Compound**	**Molecular targets**	**Observed *in vivo* effect**	**References**
AA-5-HT	FAAH (inhibition) TRPV1 (antagonism)	Anti-oedemigen and anti-hyperalgesic in the carrageenan-induced pain; analgesic in formalin-induced pain; anti-nociceptive in the CCI model; analgesic in the SNI model	Maione et al., 2007; De Novellis et al., 2008, 2011; Costa et al., 2010; Malek et al., 2016
OMDM-198	FAAH (inhibition) TRPV1 (antagonism)	Anti-nociceptive in the formalin-induced pain; anti-oedemigen in the carrageenan-induced pain; anti-nociceptive in the MIA model of pain	Maione et al., 2013; Malek et al., 2015a
Ibuprofen	COX-2 (inhibition) FAAH (inhibition)	Anti-allodynic and anti-hyperalgetic in the PNL model	Guindon and Beaulieu, 2006
ARN2508	COX-2 (inhibition) FAAH (inhibition)	Anti-inflammatory in the model of intestinal inflammation	Sasso et al., 2015

*CCI, chronic constriction injury; MIA, sodium iodoacetate; PNL, partial sciatic nerve ligation; SNI, spared nerve injury*.

### Acting on FAAH and TRPV1

Endogenous levels of AEA can activate but not desensitize TRPV1 in non-pathological conditions, leading to stimulation of pain pathways. Elevation of endocannabinoid tone by FAAH inhibitors may lead to further activation of this channel, implying that the development of combined FAAH/TRPV1 blockers may have essential therapeutic applications in directing AEA's action toward the analgesic CB_1/2_ pathway. Lack of efficacy of FAAH inhibitors in patients (as described above) may be associated with activation of the alternative AEA biotransformation pathways after completely blocking the FAAH enzyme and the ability of AEA to induce pro-nociceptive signaling through TRPV1 receptors (Maione et al., 2006; Piscitelli and Di Marzo, 2012; Starowicz et al., 2013). The most promising aforementioned agent is the endogenous lipid signaling molecule N-arachidonoyl-5-hydroxytryptamine (AA-5-HT), which was originally described as an FAAH inhibitor and called an “unique dual FAAH/TRPV1 blocker” about a decade later (Maione et al., 2007). In the context of its inhibitory activity, similar to other FAAH blockers, AA-5-HT elevates AEA levels in the CNS and on the periphery (Capasso et al., 2005; Maione et al., 2007). Moreover, AA-5-HT is able to antagonize human and rat recombinant TRPV1 receptors with an affinity slightly stronger than capsaicin, though, it is not clear whether AA-5-HT is a competitive or non-competitive antagonist (Maione et al., 2007). AA-5-HT appears to be less effective at TRPV1 blockade when the pH of the cellular environment is reduced, meaning that it may be less effective under inflammatory conditions, although this hypothesis has not been tested *in vivo* yet (Fowler et al., 2003; Paylor et al., 2006). Nevertheless, studies involving a variety of animal models have been conducted to better understand the mechanism of action of AA-5-HT. A significant contribution was completed by Maione and colleagues, who indicated the significant analgesic impact exerted by the compound in acute and chronic pain models in rodents following systemic administration (Maione et al., 2007). A subsequent study showed that intra-PAG AA-5-HT also causes analgesia and that the effect is mimicked by co-injection of URB597 and the selective TRPV1 antagonist iodoresiniferatoxin (I-RTX). Furthermore, antagonizing the CB_1_ and TRPV1 receptors abolished AA-5-HT-exerted analgesia, suggesting that these co-localized receptors are AA-5-HT molecular targets in the PAG and cortex. Moreover, single AA-5-HT dosages exhibited anti-allodynic actions more effectively than URB597 or I-RTX (De Novellis et al., 2008, 2011). In the same series of experiments, de Novellis et al. determined the role of AA-5-HT in cognitive and emotional functions (i.e., decision making, goal-directed behavior and working memory) in neuropathic pain. The authors reported that AA-5-HT is able to restore the balance between excitatory and inhibitory responses in prefrontal cortex neurons, highlighting the potential of dual-acting drugs for mitigating the central sequelae associated with neuropathic pain (De Novellis et al., 2011). AA-5-HT was also proven to dose-dependently relieve pain in a model of acute inflammation in mice after systemic administration (Costa et al., 2010). Its anti-nociceptive action was associated with an increase in AEA levels in both inflamed paws and spinal cord, though CB_1_ and TRPV1 receptors were differentially involved; TRPV1 was responsible for the anti-inflammatory properties of AA-5-HT, while both CB_1_ and TRPV1 receptors mediated its anti-hyperalgesic activity (Costa et al., 2010). Our most recent research indicates that CB_2_ receptors are also involved in the anti-nociceptive actions of AA-5-HT, after intrathecal administration (Malek et al., 2016). Despite the fact that AA-5-HT was proven to be active it is unstable due to effective hydrolysis, therefore efforts to synthesize AA-5-HT analogs with improved modes of action have been undertaken (Ortar et al., 2007). The chemical synthesis led to discovery of piperazinyl carbamates and ureas that are characterized by higher stability than AA-5-HT, nevertheless none of them have shown higher efficacy than AA-5-HT (Ortar et al., 2007, 2013; Morera et al., 2009). Of the many synthesized compounds, OMDM-198 is best described and tested *in vivo* for its anti-nociceptive and analgesic properties. OMDM-198 exhibits anti-nociceptive, anti-hyperalgesic and anti-edemic actions in acute and inflammatory pain. It inhibits the second phase of formalin-induced nociceptive behavior in rodents and blocks carrageenan-induced thermal hyperalgesia and paw edema in mice (Maione et al., 2013). The anti-hyperalgesic effects of OMDM-198 were also evaluated in an animal model of osteoarthritic pain. It was shown that the effect of OMDM-198 depends on CB_1_ receptor activation and TRPV1 receptor blockade (Malek et al., 2015a). Moreover, OMDM-198 required lower doses to achieve a comparable analgesic effect than the two single-target compounds used in the study: URB597 and SB-366,791 (TRPV1 antagonist; Malek et al., 2015a). These data suggest that the use of dual-acting compounds may be beneficial in a matter of side effects characteristic for TRPV1 antagonists (hyperthermia) and FAAH inhibitors (lack of efficacy in clinics), because of significant decrease of the working doses. It was shown that hyperthermia was not observed in animals upon AA-5-HT treatment (Maione et al., 2007; Costa et al., 2010; Malek et al., 2016). The same observations were made for OMDM-198 (unpublished data). The field of FAAH/TRPV1 blockers is still developing. A recently synthesized series of arylboronic acids characterized by high efficacy on FAAH and TRPV1 have been reported, though these hybrid compounds have not yet been evaluated in animal studies for pain (Morera et al., 2016).

### Acting on FAAH and COXs

Interactions between COX and FAAH enzymes that control endogenous lipid (including endocannabinoids) levels were reported (Guindon and Beaulieu, 2006; Jhaveri et al., 2008). AEA metabolism is of special interest because blocking FAAH enzyme activates AEA biotransformation by COX-2 pathway, leading to decreases in endocannabinoid tone. This may be another mechanism responsible for lack of analgesic action from selective FAAH inhibitors (Starowicz et al., 2013; Starowicz and Di Marzo, 2013). Indeed, co-administration of diclofenac (NSAID) and URB597 showed synergic analgesic effects in abdominal pain dependent on both CB_1_ activation and blocking prostaglandin production (Naidu et al., 2010). Another study demonstrated an additive anti-nociceptive activity of PF-3845 and diclofenac in the animal models of inflammatory and neuropathic pain (Grim et al., 2014). Moreover, ketorolac (NSAID) was shown to have an additive anti-nociceptive effect with WIN-55,212-2 (non-selective cannabinoid agonist; Ulugöl et al., 2006). In these studies, AEA levels were shown to be elevated with simultaneous inhibition of PG synthesis. Studies have shown that common NSAIDs (ibuprofen and flurbiprofen) act also as FAAH inhibitors (Fowler et al., 1999; Seidel et al., 2003; Guindon et al., 2006). The synergistic effect of ibuprofen and AEA was proven in experimental models for acute and neuropathic pain (Guindon and Beaulieu, 2006). Those data demonstrate the rationale underlying design of novel compounds that can interact with both COXs and FAAH enzymes. Indeed, numerous novel compounds with promising analgesic characteristics were developed recently, i.e., ARN2508, which was tested and effective in suppressing intestinal inflammation (Sasso et al., 2015). It also causes no gastric damage, which is often a case of chronic NSAID treatment and the protective effect was dependent on FAAH inhibition. This suggests that COX/FAAH inhibitors shouldn't exhibit NSAIDs side effects present during chronic treatment, although clinical studies would be needed to verify this hypothesis. A panel of novel ARN2508 derivatives was synthesized, of which (S)-(+)-ARN2508 demonstrated increased OEA plasma levels and decreased PGI and TXA levels after intravenous administration (Migliore et al., 2016). The *in vivo* results suggest that this compound may be useful for FAAH-COX related pathologies, such as pain.

## Summary

In this review, we have described the complex network between TRPV1 and CB_1/2_ receptors and FAAH and COX-2 enzymes, which are all involved in anti-nociceptive action of AEA. It seems that the “single-target” approach is not as effective for the AEA elevation as this has been characterized by redundancy of metabolic pathways (by i.e., FAAH, COX). Inhibition of one enzyme can activate others, leading to absence of anticipated analgesic effects. The paradigm shift from selective drugs to multi-target compounds has led to promising results in the treatment of pain, which has been summarized herein. This approach may be beneficial for the treatment of pain, which poses a difficult challenge due to the heterogeneity of its origin. Compounds, acting on more than one molecular target may have higher efficacies and better safety profiles than currently used drugs (and preclinically studied compounds) that act on a single biological target (Fowler et al., 2009; Boran and Iyengar, 2010). Acting on more than one target brings a question of selectivity of dual-acting compounds, which is of concern during the design phase (Hwang et al., 2013; Aiello et al., 2016). Moreover, as these molecules are designed to be used for chronic pain treatment it is crucial to investigate their ability to develop tolerance and (possibly) dependence, as this was not investigated yet. For the reviewed compounds no side effects were reported in the preclinical models of chronic pain, probably due to low doses necessary to obtain satisfactory pharmacological effect (analgesic, anti-nociceptive or anti-allodynic). Nevertheless, disadvantages of the use of multitarget therapy cannot be omitted and its limitation need to be considered and studied, especially when safety and efficacy in clinical trials is a major concern (Brodie et al., 2015).

## Author contributions

NM: preparing of the figure and table, writing of the manuscript, KS: review conception, writing of the manuscript.

### Conflict of interest statement

The authors declare that the research was conducted in the absence of any commercial or financial relationships that could be construed as a potential conflict of interest.

## Footnotes

^1^“Version anglaise: Minutes of the Temporary Specialist Scientific Committee (TSSC) meeting on “FAAH (Fatty Acid Amide Hydrolase) Inhibitors” of 15/02/2016 (08/03/2016).” Agence Nationale de Sécurité du Médicament et des Produits de Santé (ANSM).

## Abbreviations

2-AG: 2-arachidonoyl-glycerol
AA-5-HT: N-arachidonoyl-serotonin
AEA: N-arachidonylethanolamide, anandamide
AMG517: N-[4-[[6-[4-(trifluoromethyl)phenyl]-4-pyrimidinyl]oxy]-2-benzothiazolyl]-acetamide
ARN2508: 10r (±)-2-[3-fluoro-4-[3-(hexylcarbamoyloxy)phenyl]phenyl]propanoic acid
CB_1_: cannabinoid receptor 1
CB_2_: cannabinoid receptor 2
CNS: central nervous system
COX: cyclooxygenase
ECS: endocannabinoid system
FAAH: fatty acid amide hydrolase
I-RTX: iodoresiniferatoxin
LOX: lipoxygenase
NAGly: N-arachidonoyl glycine
NSAID: non-steroidal anti-inflammatory drug
OEA: oleoylethanolamide
OMDM-198: 3-[((chloro)pyridin-2-yl)-3-phenyl]-carbamate
PAG: periaqueductal gray
PEA: palmitoylethanolamide
PF-3845: 4-(3-(5-(trifluoromethyl)pyridin-2-yloxy)benzyl)-N-(pyridin-3-yl)piperidine-1-carboxamide
PF04457845: N-pyridazin-3-yl-4-[(3-[5-(trifluoromethyl)pyridin-2-yl]oxyphenyl)methylidene]piperidine-1-carboxamide
PG: prostaglandins
PGI: prostacyclins
SB-366, 791: (E)-3-(4-Chlorophenyl)-N-(3-methoxyphenyl)prop-2-enamide
TRPV1: transient receptor potential cation channel subfamily V member 1
TXA: thromboxanes
URB597: cyclohexylcarbamic acid 3-carbamoyl-biphenyl-3-yl ester.
